# Ozænic Pleurisies, Fœtid, Putrid, and Gangrenous

**Published:** 1909-11-06

**Authors:** G. Dieulafoy

**Affiliations:** Professor Faculty of Medicine, Member of the Academy of Medicine, Physician to the Hôtel Dieu Hospital, Paris


### Hospital Clinics.

### OZÆNIC PLEURISIES, FŒTID, PUTRID, and GANGRENOUS.

By G. DIEULAFOY, M.D., Professor Faculty of Medicine, Member of the Academy of Medicine, Physician to the Hôtel Dieu Hospital, Paris.

I shall study in this lecture all those cases of pleurisy presenting offensive and stinking fluid, and I propose to group them all under the general denomination of "Ozænic pleurisies." The dominating symptom in these cases, the symptom which immediately calls one's attention, is the extremely offensive odour of the pleural fluid, whether it be evacuated by exploratory puncture, by incision, or whether it be brought up by coughing. A few drops, removed by puncture, are sufficient to infect a whole room. I divide these pleurises into three varieties: (*a*) Fœtid, (*b*) Putrid, and (*c*) Gangrenous.

### Fœtid Pleurisies.

This denomination must be reserved to those cases in which the offensive odour of the fluid is due to neither putrefaction nor gangrene of the tissues. This variety of pleurisy is not putrid, because the fluid has none of the characteristics presented by liquids due to putrefaction; it does not give off any gas in the pleural cavity, and there is therefore no concomitant pneumothorax; on the other hand, inoculated subcutaneously to animals, it does not give rise to emphysematous cellulitis; cultivated, it does not produce fermentation or gas. Furthermore, it is not gangrenous, because the fluid does not contain any fragments of sphacelated tissue, nor do the walls of the pleural cavity present any trace of gangrene. These differences have already been noted by Laennec and Trousseau.

Sero-purulent and purulent pleurisies with fœted effusion are quite frequent. The patient whose case I related in detail in one of my lectures on interlobar pleurisy had a fœtid pleurisy which was neither putrid nor gangrenous; the sputum and the breath were fœtid, the pus removed by operation was fœtid, but there was no fermentation with formation of gas within the chest, and the operation showed that there was no trace of gangrene. I have also related the case of a little girl suffering from interlobar pleurisy in the left side, whom I saw in consultation with the late Professor Potain and Dr. Simon; her sputum and breath were both extremely fœtid. Empyema with excision of a rib, performed by Dr. Tuffier, gave issue to very offensive pus. There was, however, neither fermentation nor gangrene.

In two cases of purulent mediastinal pleurisy which I described in a former lecture the vomica was fœtid. It is worthy of notice that if purulent inflammation of the general cavity of the pleura is susceptible of giving rise to fœtid effusion, this is the case much more frequently in encysted pleurisy. In the former case the inflammation has more tendency to be putrid or gangrenous.

The fluid, in fœtid pleurisy, is sero-purulent and contains different varieties of ærobic and anærobic germs (pneumococcus, streptococcus, bac. coli). There are also micro-organisms which do not give rise to either gangrene or putrefaction, but which can produce fœtid odours, just as certain germs can produce colouring matter. In order to make a diagnosis in these cases one must not simply consider the odour only. They are distinguished from putrid pleurisies by laboratory methods and from gangrenous pleurisies by the absence of sphacelated tissue in the effusion. Although they are less serious than either of these two last varieties and produce much less rapidly collapse and tendency to syncope, one must lose no time when dealing with them. Thoracotomy, with or without excision of one or more ribs, must be performed as soon as possible.

### Putrid Pleurisies.

These differ from the foregoing by the presence of the symptoms of putrefaction; formation of gas in the tubes in which the liquid is received, and also in the pleural cavity, thus giving rise to pneumothorax, development of emphysematous cellulitis in the chest walls after puncture with the needle; gaseous œdema or cellulitis when the liquid is injected into an animal; gravity of the patient's condition with extremely rapid and small pulse, tendency to collapse and syncope. Here are a few cases taken from my clinical notes:—

Woman, æt. 32, admitted into the wards on the 24th of October, 1899, in a most alarming state. High fever, pallid face, violent dyspnœa, great prostration with impending collapse, marked emaciation. When near the patient one was immediately struck by the fœtor of the breath; she coughed a good deal and expectorated grey, sanious, and very offensive matter. One might have suspected gangrene of the lung. The examination of the chest showed, however, the presence of an effusion of about 500 or 600 grammes on the right side, with wet rales above it. This pleuro-pulmonary affection had begun suddenly on the 16th of September by a sharp pain in the side and rigors. The patient was at that time convalescent from a serious operation; on the 19th of August her womb and ovaries had been removed on account of double salpingitis and fibroma of the uterus. The operation had been followed by serious hæmorrhage which brought on syncope, and which left the patient in such a weak state that for several days she was hardly aware of what was going on around her. She recovered, however, and left the hospital on the 10th of September. The actual malady began six days later—that is four weeks after the operation. She began to cough and to bring up fœtid expectoration. Dr. Appert saw her and made a puncture which gave issue to half a litre of dirty, but non-fœtid, liquid. As there was no improvement she was nt to the Hôtel Dieu, where a second puncture gave issue to greyish, sanious liquid, so fœtid that one could not but help thinking of gangrene.

The above facts showed plainly enough that this woman had a pulmonary lesion with fœtid sputum and pleural effusion, and, moreover, that the lung trouble had preceded the effusion, for the sputum had been fœtid before the effusion had become so. The nature and origin of the lesions remained to be determined. The question was: Was there gangrene of the lung and pleura, or was it a case of putrid infection without mortification of the tissues, which is a very different thing from the prognostic point of view? Furthermore, what was the point of entrance of the infection?

Was it due to embolism, or had it an aerial origin? Had the germ penetrated through the blood-stream or through the air?

The operation performed four weeks before might cause one to suspect that the pleuro-pulmonary lesions were due to abdominal embolism. It is quite true that a certain time had elapsed since the operation and that the result of the latter had been favourable; but it is well known that distant embolic infection may supervene several weeks after the disappearance of the primary focus. Examples of this are found in appendicitis in which infection of the liver, of the lung, of the pleura, may take place after recovery from the primary affection. In otitis, abscess of the cerebrum or of the cerebellum, or pulmonary gangrene, may supervene after the disappearance of the ear trouble. The same may occur after even quite a normal confinement, in which all the ordinary antiseptic precautions have been taken. The pleuro-pulmonary lesions of this patient, however, were not due to embolism. In her answers to our questions she had informed us that for about a month she had had a fœtid vaginal discharge, which had not been modified by daily injections. The examination of the vagina with the finger and speculum showed the presence of a greyish mass, which turned out to be a tampon of gauze left there by mistake after the operation. This gauze had a most offensive odour, and after its removal about 50 grammes of very fœtid liquid ran out. After thorough and repeated lavage we found on the mucous membrane at the bottom of the vagina several ulcerations, the floors of which were sanious and the edges red and bleeding readily. It was through these ulcerations that the germs had penetrated in the blood, which had conveyed them to the lung, where they had produced the lesions existing in this patient.

After the gauze had been removed the temperature went down, but the general state remained bad and the temperature soon went up again, for the pulmonary lesions continued their evolution. Operative intervention was therefore necessary, and was performed by Mr. Marion, assistant surgeon to the hospital. Half a litre of very fœtid liquid was removed, but it did not contain any trace of sphacelated tissue. There was therefore no gangrene of the pleurum, nor was there any pulmonary gangrene, for the sputum contained neither elastic fibres nor sphacelated matter. The operation was fully successful, the temperature went down, the general state improved rapidly, and three weeks later the patient had quite recovered. Bacteriological examination of the vaginal liquid, of the pleural effusion, and of the sputum showed the presence of identical germs. Cultures in bouillon and on gelose gave rise to the formation of gas, which proved that there was putrid fermentation. Experiments on guinea-pigs and rabbits, both with the vaginal and pleural pus, gave only insignificant results.

### Some Conclusions.

We can sum up the above interesting case as follows: Bacteriological examination has enabled us to determine the nature and the evolution of the infection from which our patient suffered. The affection passed through two stages. It was from the bottom of the vagina, transformed into a closed cavity by the gauze, that the toxi-infection started. Dr. Hallé has shown in a very interesting work that the vagina contains normally ærobic germs in the form of streptococci differing from the S. pyogenus, and also germs which are strictly anærobic (bacilli), and inoculated into animals may give rise to abscesses and gangrene.

In our patient the infection, of anærobic nature, started in the vagina. The germs were conveyed through the nervous system to the right heart, and thence were thrown into the pulmonary circulation. They then passed into the pleurum unless the latter became infected separately. The pulmonary infection produced rigors, fever, and pain in the side. A certain area of the lung became embolised and transformed into a putrid but non-gangrenous infarct. The pleural effusion, slightly streaky at the first puncture, became putrid at the second. We have thus successively the vaginal, pulmonary, and pleural stages of the infection, the clinical case presenting the exactness of an experiment.

Throughout the entire evolution the infection was a putrid one and not gangrenous. This is proved, first, by the formation of gas in anærobic cultures, and secondly, by the absence of sphacelated matter in the effusion and sputum, also by the absence of elastic fibres in the latter. In this case mortification was not associated with the process of putrefaction.

Ozænic pleurisies are frequent after appendicitis. I have notes on a number of cases, and I will give you a brief account of some of them. A patient was admitted into my wards in a dying condition. He had extensive right pleurisy with pneumothorax. He died in a few hours before any operation could be done. The history of the case was given by his relatives, and the post-mortem enabled us to understand the whole process. The pleural cavity contained $3\frac{1}{2}$ litres of stinking pus and also gas. Both the pleurisy and pneumothorax were the result of appendicular infection. The lesions had started in the appendix, spread into the right side of the abdomen and gained the right thoracic cavity. If the putrid infection had not an embolic origin, as in the case related above, the lesions must have spread by continuity.

A man was taken suddenly with a sharp pain in the left side, and the dyspnœa was so violent that he had to be taken to the nearest chemist's shop, where an injection of morphine brought relief. The dyspnœa, however, soon reappeared, together with a hacking cough, and the general state became very bad. He was then admitted into Dr. Widal's wards. The physical symptoms of effusion with pneumothorax were found to be present in the left side. By immediate puncture 1 litre of puriform and very fœtid liquid was removed. At the point of puncture emphysematous cellulitis developed. The patient died before resection, which had been decided upon, could be performed. A large quantity of sanious and fœtid liquid mixed with gas was found in left pleural cavity. The walls of the chest were lined with fibrinous membranes without any trace of gangrene either of the pleurum or lung. The germs found to be present were the proteus vulgaris. One cubic centimetre of the effusion inoculated to a guinea-pig gave rise to an abscess with formation of gas. The abscess ulcerated and gave issue to a sanious pus containing various germs, principally proteus vulgaris.

### Other Cases.

A man, æt 23, admitted into Dr. Courtois-Suffit's wards at Beaujou Hospital with fever, rigors, and sharp pain at the base of the right lung, with signs of pleural effusion. A day or two later he had dyspnœa and cyanosis. On puncture 900 grammes of purulent and fœtid liquid were removed. Two days later emphysematous cellulitis set in at the point of puncture. The patient got worse and worse every day. Violent dyspnœa, abundant perspiration; temperature 103°-104°. Operation was performed, with evacuation of a large quantity of fœtid pus; the cavity was washed out with solution of permanganate of potash. In spite of all, the patient died. Post-mortem: Fœtid liquid in pleural cavity, serous lining thickened, but no trace of gangrene anywhere.

A robust man of 42 was suddenly taken with rigors, cough, and pain at the base of the left lung. After a fortnight he entered Dr. Boinet's wards at Marseilles with signs of pneumothorax. Operation gave issue to 2 litres of fœtid pus and stinking gas. Death 12 days after operation. Post-mortem: Pleural cavity divided into three compartments containing liquid and gas. There was no communication with the lung. No tuberculous lesions, no gangrene either pulmonary or pleural.

A young doctor had right pleurisy; he was punctured, and fœtid sero-purulent liquid removed. The liquid reformed rapidly, and at a second puncture fœtid pus appeared. The latter, examined microscopically, showed presence of various germs. Cultures gave staphylococci. His general state got worse, and emphysematous cellulitis developed at points of puncture. At operation large quantity of fœtid pus and gas escaped, and the patient recovered. In Dr. Achard's words, "this is a case of putrid pleurisy without gangrene; no gangrenous focus had been made out clinically, and the evolution of the disease resembled in nothing that of pleuro-pulmonary gangrene."

Let us now draw the general conclusions from the above cases. Putrid pleurisies occupy most frequently the general pleural cavity; fœtid pleurisies, on the contrary, are generally encysted (interlobar, mediastinal, diaphragmatic). In putrid pleurisies the liquid is sero-purulent, thin, greyish; placed in a test-tube it forms two layers, a lower one, thick, opaque, and an upper one more transparent. They frequently have an embolic origin, the primary focus varying considerably: vaginal or appendicular, as in the cases described above; otitis, osteomyelitis, phlebitis, etc.

In certain cases the pleurisy is due to neighbouring lesions (lung, mediastinum). It may follow abdominal lesions, suppuration of kidney or liver, subphrenic abscess with or without perforation of the diaphragm. In other cases the cause and origin remain unknown. Under the influence of germs, especially of the anærobic variety, putrid pleurisies have as a special attribute the formation of gas (putrefaction), which explains the appearance of pneumothorax without perforation of the pleural walls. This variety of pneumothorax, formerly called "essential," is therefore quite different from that due to perforation with penetration of atmospheric air. From a clinical point of view, pneumothorax due to putrefaction gives, on auscultation and percussion, the same symptoms as pneumothorax by perforation: tympanitic resonance, amphoric murmur, metallic respiration, Hippocratic succussion.

Formation of gas may take place in the thickness of the chest walls themselves and give rise to emphysematous cellulitis. A simple exploratory puncture may sow the germs in the tissues, and a few hours later emphysematous cellulitis is the consequence. Experiments on animals give identical results. A drop of the pleural effusion inoculated in the subcutaneous tissue gives rise to emphysematous cellulitis (Widal). Finally, gas may be obtained in anærobic cultures made with the germs of these pleurisies.

The diagnosis of putrid pleurisy is impossible without puncture. It is quite true that there are very serious general symptoms (very small and rapid pulse, dyspnœic rigor, prostration, tendency to collapse) which are rare in other varieties of pleurisy, and if besides this pneumothorax develops, putrefaction should be suspected.

Nevertheless, the only certain way of ascertaining the putrid nature of the effusion is by an exploratory puncture, and whenever there is the slightest doubt about this one must not hesitate to puncture, even when the effusion is very small in amount. Moreover, the puncture must immediately be followed by thoracotomy with or without excision of a rib, for the track of the needle in the tissues may rapidly become infected, and in the space of a few hours give rise to emphysematous cellulitis. It may be objected that the puncture only shows whether the effusion is fœtid or not, and gives no information as to whether it is putrid. My answer is that it does not matter for the moment whether the pleurisy be a fœtid or putrid one, for all ozænic pleurisies must be operated without loss of time; bacteriological and experimental investigation comes only after the operation.

Gangrenous Pleurisies.

These, like putrid pleurisies, present the characteristics of putrefaction. What distinguishes them is not the fœtor of the effusion but the mortification of the tissues, which shows itself by the presence of sphacelated matter in suspension in the liquid or adhering to the inner side of the chest walls.

Gangrenous pleurisy has many symptoms in common with the putrid variety, and presents two anatomo-pathological forms. In the first the gangrene is limited to the pleura, and the lung is not affected; in the second there is simultaneously pleural and pulmonary gangrene. The latter is the most serious. I shall give you a few brief notes on cases of gangrene limited to the pleura.

A child, æt. 11, suffered from left pleurisy, and for a few days the symptoms were not very marked, the dyspnœa being only slight. One night the child woke up suddenly, cried, and presented extremely violent dyspnœa. Drs. Comby and Vogt were called, found a temperature of 104° and symptoms of a pleural effusion, with pneumothorax on the left side, which they thought might be due to a perforation of tuberculous nature. Four days later a puncture gave issue to half a litre of horribly fœtid pus. The diagnosis of gangrenous pleurisy was made. Dr Comby operated and removed a mass of sphacelated membranes and two or three litres of stinking pus. After many ups and downs the little patient recovered.

A man of phthisical appearance, suffering from pneumothorax, was admitted into Dr. Render's wards at the Hospital Necker. He was emaciated, had great dyspnœa, coughed a great deal, but had no fever, and his breath was not fœtid. He presented the physical symptoms of effusion and pneumothorax in the right side. The affection had begun a few days before by a violent pain in the side. Two days after his admission to the hospital he got much worse; there was violent oppression, cyanosis, and the heart was pushed over to the left. An exploratory puncture brought very fœtid pus. Dr. Rist removed three-quarters of a litre of fœtid pus and washed out the cavity with a solution of permanganate of potash. Two days later emphysematous cellulitis developed at the right base of the thorax; the knife gave issue to fœtid serum mixed with bubbles of gas. A little later emphysematous cellulitis appeared in the left arm, where the patient had been recently vaccinated. The state of the patient remained very serious: there was much agitation, with delirium; the pus flowing from the chest became again very fœtid, and one day, while a lavage of the cavity was being made, a large mass of black sphacelated and extremely offensive matter came away, in which the microscope showed the presence of elastic fibres. The patient improved, and eventually recovered. Dr. Rist examined the matter bacteriologically and found, principally, anærobic germs present.

In both the above cases there was gangrenous pleurisy without gangrene of the lung. There are cases, as I have said before, where there is pulmonary gangrene at the same time. Generally, in these cases, the disease begins by gangrene of the lung. These facts are well known, and it is not necessary to insist any further on the matter, for the pathogeny of pulmonary gangrene applies also to pleuro-pulmonary gangrene, the origin being either embolic (otitis, appendicitis, etc.) or aerial.

From the point of view of diagnosis gangrenous pleurisies resemble in part those of the putrid variety. The violence of the pain in the side, the fever, the weakness and smallness of the pulse, the pallor of the face, the great prostration, the tendency to collapse show clearly enough the extreme gravity of gangrenous pleurisy; the fœtor of the breath, the stinking and bloody sputum, containing elastic fibres, prove the participation of the lung in the gangrenous process.

Gangrene marks a further stage in the gravity of putrid pleurisies, although these two varieties are closely related to each other. The same ærobic or anærobic germs which have produced a simply putrid pleurisy may determine gangrenous lesions, either in the patient himself, or, by inoculation, in animals. In Dr. Widal's case the putrid pleurisy was not gangrenous—this was verified at the post-mortem—and yet the pleural liquid inoculated to a guinea-pig gave rise to emphysematous cellulitis, and the animal died with extensive sphacelus of the skin of the abdomen and chest. In Dr. Render's case the pleurisy was, at first, of the putrid variety; later on, as if a further evolution of the process had taken place, a large piece of gangrenous matter was removed from the pleural cavity. Between putrid and gangrenous pleurisies it is very difficult to draw an absolute line; putrefaction and mortification may develop simultaneously or successively.

I have now given you a description of ozænic pleurisies, with their three varieties—fœtid, putrid, and gangrenous. The fœtor of the effusion, which constitutes the first element of the diagnosis, can only be discovered by an exploratory puncture, which ought to be made as early as possible. Immediate surgical intervention is the only treatment.

